# Keystone Veterinary Medical Association

**Published:** 1890-10

**Authors:** 


*Keystone Veterinary Medical Association.*—The annual meeting of the Keystone Veterinary Medical Association was held at the College of Physicians, September 6th, the President, Dr. Huidekoper, in the chair. Drs. Magill, Maurice, Huidekoper, Hoskins, Lusson, Weber, Kooker, Ridge, Bridge, Goentner, Harger and Eves were present. The minutes of June meeting read and approved.

As none of the board of trustees were present, their report was ordered to be made in writing at the next meeting. The Secretary made the following report of the proceedings of the year.

SECRETARY'S REPORT.

The Keystone Veterinary Medical Association has thirty-four members and one honorary member. It had nine meetings during the year; average attendance per night was fourteen; average meetings attended by each member, were 33⅓ per cent. Dr. Magill has attended every meeting since he joined. Five new members were taken in during the year; three members sent in their resignations. No names have been stricken off during the year. Drs. Hickman, Montgomery, Maurice and Shaufler were not present during the year. There was no meeting in September, and the election of officers was held in October. In November, Dr. Hoskins read a paper on "Osteo Porosis," and Dr. Weber reported a case. In December Dr. Lintz read a paper on "Influenza," and Dr. Felton reported a case. In January, Dr. Ridge read a paper on "Parturient Apoplexy." Dr. Harger, who was to report a case, was absent. In February, the Association changed its place of meeting from the Veterinary Department, University of Pennsylvania, to the Hall of the College of Physicians. Dr. Montgomery, the essayist was not present; Dr. Lusson reported a case. In March, Dr. J. B. Raynor gave a committed essay on "Dystokia," and Dr. Goentner reported a case of rabies. In April, Dr. Goentner, the essayist, was not present; Dr. Maurice, the reporter, was also absent. In May, Dr. Miller, essayist, was absent on business, and Dr. Hartman, reporter, sent no excuse. Dr. Huidekoper read a paper on "Fever." June, the last meeting of the year, was occupied with reports of cases.

Taking the year's work as a whole, we think it poor; only five papers and four reports of cases in nine meetings, is certainly very bad showing for thirty-four members. But there is one thing that made the meetings interesting, that is, the voluntary articles; there was no meeting but where some one was prepared to take the part of the absentee, and the meetings are always pleasant to attend.

There were four charges of breach of code of ethics during the year. They were all settled satisfactorily, with no penalties.

*List of Attendance.*—Drs. Miller, 1; Zuill, 5; Goentner, 3; Glass, 4; Hoskins, 8; Huidekoper, 6; Weber, 4; Keelor, 1; J. B. Rayner, 3; T. B. Rayner, 4; Kooker, 7; Bridge, 3; Vandegrift, 2; Williams, 5; Montgomery, 0; Lintz, 5; Hickman, 0; Cullen, 3; Webster, 5; H. Formad, 1; Eves, 5; Harger, 5; Ridge, 8; Felton, 4; Maurice, 0; Hartman, 2; Lusson, 4; Robert Formad, 2; Magill, 7; Werntz, 4; Bachman, 1; Mattson, 1; Birch, 1; Shaufler, 0.

W. H. Ridge, *Secretary.*

Dr. Goenter made his annual report, stating that there was $20.49 in the treasury, and a number of uncollected dues. Drs. Magill and Lusson were appointed auditors. The secretary read the resignation of Dr. T. B. Rayner, which was referred to the board of trustees. Dr. Vandegrift, the essayist, was not present.

The election of officers resulted in the choice for President, W. H. Hoskins; Vice-President, W. H. Ridge; Secretary, C. H. Magill; Treasurer, Charles T. Goentner; Trustees, Weber, Chairman; Kooker, Lusson, Eves, Harger.

Dr. Magill reported the following case: Gray gelding, 15 years old; weight, about 1350 pounds. Was called March 21st. *History.*—It has been standing in stall for about one week, lame in one leg—from thrush, they thought—and they treated it accordingly, but it did not get well. March 27th.—They put him to a truck, weight about 1800, then loaded him with about 3000 pounds additional, drove him to depot and backed him up to platform, and as he struck it he went down, and it took several men to raise him up. They put him to wagon, and drove him to another part of the depot, and as they turned him around to back up again, he fell a second time, and with great difficulty they got him up and to the stable. By the time he got to the stable he was in a profuse sweat. The next morning, the 21st instant, found him in stall, eating hay; seemed bright; pulse, 60; respirations, 18; temperature, 101 2-5°. Moved him out of the stall with great difficulty, and when he walked he staggered behind, and seemed as if he did not know where he was putting his hind feet, which knuckled over. *Examination per Rectum.*—Very sensitive at the lumbo-sacral articulation; urine normal. No loss of sensation in gluteal muscles. *Diagnosis.*—Strain of lumbo-sacral articulation. The trouble of the hind leg being due to some pressure on spinal cord. The prognosis unfavorable. I put him in slings, and gave him a ball of aloes, also a diuretic. Let him stand in slings until April 11th, when I called to see him. Found the slings broken, the horse down and much worse. Advised destruction. The autopsy showed the internal organs normal, but the articulations of hind legs ulcerated. The lumbo-sacral articulation very much inflamed, rough and fibrinous, lead colored in spots; a congestion of the heads of the last three ribs on right side. The ribs were very brittle. The spinal cord was soft, and there was a quantity of fluid in spinal column. The jaw was very thin. The result of the autopsy, Dr. Magill thinks, revealed osteo-porosis, which was, undoubtedly, the cause of the lameness at the start, and when the horse had backed up to the platform, the load striking hard against it, pushing his hind legs under him, he loosened the lumbo-sacral articulation, and caused pressure on the cord. It also was the cause of the soft condition of the cord.

*Discussion.*—Dr. Huidekoper cited a case which first seemed like a case of incomplete fracture of the tibia. Later, the leg swelled up, the tendo-Achilles was swollen and sensisive, but soon improved. After about a month's use, he called again, when he found the jaw thickened and diagnostic symptoms of osteo-porosis. He believed both the first lameness and the inflammation of the tendons to have been symptoms of the disease.

Dr. Hoskins, the new president, now took the chair, and made an address, warning the association against future delinquencies.

The special Committee on Tumors was not prepared with sections, which were in possession of Dr. Formad, absent. Dr. Ridge brought sections. The committee stated the tumor to be a sarcoma. Dr. Ridge's sections were those of a fibroma. The committee were again instructed to bring their sections at the next meeting, and to bring what remained of the tumor for macroscopic examination. Dr. Goentner reported a case of amputation of the uterus in a cow. The case has done well. Dr. Lusson reported a case of laminitis following parturition. Dr. Bridge stated he had seen it often. Dr. Eves also stated he had a case of it at present, which occurred twenty-four hours after parturition. Dr. Bridge reported some cattle at Bristol, Bucks County, which had Texas fever. Fifteen head died; they had been brought from Ohio.

Dr. Eves reported a local outbreak of disease in cattle in Delaware. It extended in a territory about one mile long and one-half wide, some thirty cows dying in a period of six weeks; they had partial paralysis of hind parts; great purging; temperature 101½°; seemed very dull; urine normal. As a dog had bitten these animals, and the animals constantly licked the spot where they were bitten, with other symptoms, he made a diagnosis of rabies.

Dr. Goentner reported a case in an ox occurring eleven days after being bitten. Dr. Bridge reported a case fifteen days after being bitten. A question came up regarding the normal temperature of cattle. Dr. Bridge said he thought 102° F. the average. Dr. Hoskins reported a case where a cow acted strangely, pushing its head against objects until she had the head denuded of hair and skin. He chained her. Next morning found her head greatly swollen. She was still acting wildly; ordered her killed; bowels and all internal digestive organs normal; cerebrum contained clots; the medulla inflamed intensely; the meninges were also inflamed. Dr. Hoskins thought this might have been a case of rabies, but Dr. Bridge thought it probably some sporadic brain trouble. Dr. Goentner said he would not give a diagnosis of rabies unless perfectly sure of the diagnosis, but would give diagnosis of some other trouble if in doubt. The next case has been before the Association since the April meeting, Dr. Hoskins' horse "Billy." Dr. Hoskins had invited the members of the Association several times to come and examine him, which several did, with varying results. The symptoms have already been recorded in THE JOURNAL OF COMPARATIVE MEDICINE AND VETERINARY ARCHIVES.

In April Dr. Hoskins attended an outbreak of glanders in a stable having one of the animals brought to his stable. The stable was thoroughly disinfected after the horse was taken out; then Dr. Hoskins' horse was put in. In about two weeks found animal ailing; temperature 103° F.; respiration normal; pulse ditto; did not eat. There soon appeared an abscess on the side (over the ribs). On opening it, a yellow, sticky serum escaped. The submaxillary gland on one side became enlarged. This condition lasted about three months. Several veterinarians examined the animal, and, up to June meeting, all but one made the diagnosis of glanders. At June meeting the invitation was again extended for members to examine him. Among those to examine him was Dr. Weber, who made a diagnosis of chronic pleurisy, following, most likely, influenza. At this time there was no fluid in the thoracic cavity, but it soon began filling. The temperature varied all through the course of the disease; some days it would be normal, perhaps the next morning there would be a temperature of 105° F. This continued until July, when he died. *Autopsy:* The organs in the abdominal cavity were normal; the thoracic cavity contained about forty quarts of fluid; the lungs were so much disorganized that it seemed miraculous that he lived so long. There were no signs of glanders anywhere, the only lesions were those of chronic pleurisy. Dr. Kooker had also given it as his opinion that the animal was not suffering from glanders, but synovitis. This was in the first stage, which was also correct.

This case is instructive in two points : First, that we should never take a thing for granted, but make a thorough examination, which takes time Second, that it is very difficult to diagnose glanders without all of the characteristic symptoms.

Dr. Hoskins thinks it a very bad practice giving a purgative in suspected cases of glanders, for if it happens to be some disease like a case of influenza, it will lessen the chances of recovery, as it did in this case. Dr. Bridge advocated the importance of inoculating guinea-pigs. Dr. Bridge read the law governing glanders in this State, the law of May 9th, 1889, as follows :

NO. 167.—STATUTES OF PENNSYLVANIA SESSION, 1889.

*An Act to Prevent the Spread of Contagious Diseases among Domestic Animals.*

SECTION I.—*Be it enacted, etc.*, That when it shall be brought to the notice of the Secretary of the State Board of Agriculture that any contagious disease, not otherwise provided for by law, prevails among domestic animals, he may take such measures to prevent its spread as may be deemed expedient, and for this purpose shall have power to place infected animals, herds, buildings and farms in quarantine, and to prevent the movement of animals or objects likely to convey the contagion, except upon proper permits, and with the consent and approval of the governor ; to make such rules and regulations for the government of such quarantine as may be deemed necessary to effectively carry out the purpose of this act.

SEC. 2. That any person or persons who shall wilfully or intentionally interfere with any officer or officers duly authorized to carry out the provisions of this act, or shall wilfully or intentionally violate the provisions of the quarantine authorized by Section 1 of this act, shall be deemed guilty of a misdemeanor, and upon conviction shall be liable to an imprisonment not exceeding three months, or a fine not exceeding one hundred dollars, or both, at the discretion of the court.

SEC. 3. That when it shall be necessary or expedient to kill any animal or animals to prevent the spread of contagious diseases, it or they shall first be appraised by sworn appraisers, who shall have due consideration for the actual condition of the animal or animals at the time of appraisement, and the owner or owners shall be entitled to receive from the Secretary of the State Board of Agriculture a certificate of value, which may be paid from current appropriations, or by a subsequent appropriation by the legislature. *Provided*, That the amount of such certificates, issued in any one year, shall not exceed the sum of twenty-five dollars.

SEC. 4. That for the economical eradication of contagious diseases of domestic animals, the Secretary of the State Board of Agriculture shall have power, with the consent and approval of the governor, to arrange for and carry into effect terms of coöperation with the proper officers of the national government.

SEC. 5. That all acts or parts of acts inconsistent herewith are hereby repealed.

Approved the 9th day of May, A. D. 1889.

JAMES A. BEAVER.

Dr. Bridge then added :

You will perceive that the amount appropriated for the destruction of animals affected with contagious diseases is very limited, when you take into consideration the size of the State and the number of domestic animals this act is supposed to cover. This act does not provide for "contagious pleuro-pneumonia," as that is otherwise provided for (fortunately we have none of this disease in the State that we know of), and it is to be hoped that we may never have it again, as it would be impossible to kill all animals affected with contagious diseases and pay for them with this limited sum, Mr. Edge, the Secretary of the State Board of Agriculture, has determined to use the major part of the appropriation for the suppression of "glanders." For with this small appropriation it would be useless to attack hog cholera, or tuberculosis. There has been quite a number of horses and mules killed affected with glanders and farcy under this law. The appraisements up to the present have not exceeded thirtv dollars per capita, and the owners so far seem to have been perfectly satisfied. If the appraisement at any time should exceed this sum I do not know whether it would meet the approval of the Governor or not, for the last legislature passed an appropriation for animals killed affected with glanders, which appraisers had valued at two-thirds of their healthy price. The Governor vetoed this bill, giving as his reason for so doing that glandered horses had no value, and that the appraisement was excessive. The Governor is perfectly right in one sense, but it is policy to pay a fair price for these animals, as then the owners would report them to the Board of Agriculture, whereas if they are appraised as valueless, persons owning such animals will be tempted to sell or trade as often as they can to advantage ; thus the disease would be spread in diverse places, and the cost of the destruction of animals thus infected would be far in excess of what the cost would be if the State would pay a good price, or as much as the owners could get for them from any other source. This was the policy which the secretary adopted when he first began the extirpation of contagious pleuro-pneumonia (paying a good price for all diseased stock). He found it cheaper to have the disease reported to him than to pay a large veterinary force to hunt it up when concealed, and you know how satisfactory this policy has worked. It has cost less for the extirpation of "C. P. P." in Pennsylvania than in any other State, and I know that Mr. Edge will exercise as liberal a policy in dealing with glanders as the Governor will allow. All owners of glandered horses, and all veterinarians, who know of such stock, should report to him at Harrisburg, Pa.

WILLIAM H. RIDGE, *Secretary.*

